# PP82 Prevention And Intervention Software Applications For People At Risk Of Suicide: Effectiveness And Safety

**DOI:** 10.1017/S0266462324002514

**Published:** 2025-01-07

**Authors:** Amado Rivero-Santana, Andrea Duarte-Díaz, Yolanda Álvarez-Pérez, Renata Linertová, Alezandra Torres-Castaño, Himar González-Pacheco, Carmen Guirado-Fuentes, Analía Abt Sacks, Vanesa Ramos García, Ana Toledo-Chávarri, Francisco Javier Acosta-Artiles, Pedro Serrano-Pérez, Carme Carrion, Marta Guillén, Lilisbeth Perestelo-Pérez

## Abstract

**Introduction:**

Suicide poses a severe public health challenge worldwide, impacting individuals, families, work, and society. The multifaceted nature of suicide demands a complex approach involving psychological, biological, social, cultural, and environmental factors. Recognizing suicide’s status as the leading external cause of death in Spain, prevention increasingly incorporates technology, specifically mobile and software applications.

**Methods:**

A systematic review of the effectiveness and safety of mobile and software applications was conducted (MEDLINE, Embase, CINAHL, and PsycINFO databases). Outcome variables included: suicide; suicidal behavior; suicidal intent; suicidal ideation/thinking; self-perceived suicide risk; using/seeking mental health services; associated mental symptoms; mental health-related quality of life; satisfaction of the user and the health professional; adverse events related to the app, as defined in the included studies. Studies that do not include suicidal behavior, intention, or ideation were excluded. Where available data allowed, a meta-analysis was conducted for each outcome variable.

**Results:**

One systematic review and 13 randomized controlled trials (n=2,952) were analyzed. No significant differences were found in deaths by suicide or suicide attempts. At post-intervention, small but significant reductions were observed in suicidal ideation, hopelessness, depression, and worry, with anxiety reduction slightly above statistical significance. At follow-up (8 to 52 weeks), these variables also obtained significant results, except depression and suicidal ideation. Regarding safety, there was no significant difference in safety phone calls for participants with suicidal ideation.

**Conclusions:**

The evidence on suicide prevention app effectiveness is of low quality, precluding conclusive findings. Attempt reduction is suggested at 21 percent, but the confidence interval includes a potential 60 percent increase. Evidence on suicide-related psychological variables (suicide ideation, depression, hopelessness, and anxiety) is of higher quality (low–moderate), but effects are small and clinically uncertain. Safety findings are uncertain, impacting risk/benefit balance.

